# Filament Compliance Influences Cooperative Activation of Thin Filaments and the Dynamics of Force Production in Skeletal Muscle

**DOI:** 10.1371/journal.pcbi.1002506

**Published:** 2012-05-10

**Authors:** Bertrand C. W. Tanner, Thomas L. Daniel, Michael Regnier

**Affiliations:** 1 Department of Molecular Physiology and Biophysics, University of Vermont, Burlington, Vermont, United States of America; 2 Department of Bioengineering, University of Washington, Seattle, Washington, United States of America; 3 Department of Biology, University of Washington, Seattle, Washington, United States of America; University of California San Diego, United States of America

## Abstract

Striated muscle contraction is a highly cooperative process initiated by Ca^2+^ binding to the troponin complex, which leads to tropomyosin movement and myosin cross-bridge (XB) formation along thin filaments. Experimental and computational studies suggest skeletal muscle fiber activation is greatly augmented by cooperative interactions between neighboring thin filament regulatory units (RU-RU cooperativity; 1 RU = 7 actin monomers+1 troponin complex+1 tropomyosin molecule). XB binding can also amplify thin filament activation through interactions with RUs (XB-RU cooperativity). Because these interactions occur with a temporal order, they can be considered kinetic forms of cooperativity. Our previous spatially-explicit models illustrated that mechanical forms of cooperativity also exist, arising from XB-induced XB binding (XB-XB cooperativity). These mechanical and kinetic forms of cooperativity are likely coordinated during muscle contraction, but the relative contribution from each of these mechanisms is difficult to separate experimentally. To investigate these contributions we built a multi-filament model of the half sarcomere, allowing RU activation kinetics to vary with the state of neighboring RUs or XBs. Simulations suggest Ca^2+^ binding to troponin activates a thin filament distance spanning 9 to 11 actins and coupled RU-RU interactions dominate the cooperative force response in skeletal muscle, consistent with measurements from rabbit psoas fibers. XB binding was critical for stabilizing thin filament activation, particularly at submaximal Ca^2+^ levels, even though XB-RU cooperativity amplified force less than RU-RU cooperativity. Similar to previous studies, XB-XB cooperativity scaled inversely with lattice stiffness, leading to slower rates of force development as stiffness decreased. Including RU-RU and XB-RU cooperativity in this model resulted in the novel prediction that the force-[Ca^2+^] relationship can vary due to filament and XB compliance. Simulations also suggest kinetic forms of cooperativity occur rapidly and dominate early to get activation, while mechanical forms of cooperativity act more slowly, augmenting XB binding as force continues to develop.

## Author Summary

In striated muscle myosin binds to actin and converts chemical energy from ATP hydrolysis into force, work, and power. Myosin cross-bridge binding is regulated by Ca^2+^ and the thin filament proteins troponin and tropomyosin. Cooperative interactions between actin, myosin, troponin, and tropomyosin greatly influence spatial and kinetic properties of thin filament activation, thereby affecting muscle mechanics and contractility. Such cooperative interactions are complex and individual contributions from the different contractile and regulatory proteins are difficult to separate experimentally. However, a few theoretical models have explored interactions between the spatial, kinetic, and mechanical processes that affect cooperative cross-bridge binding to actin. Building on our prior spatially-explicit computational models, we investigated the relative contributions of thin filament regulatory proteins and cross-bridges to cooperatively amplify skeletal muscle force production. We find that Ca^2+^-dependent contraction in skeletal muscle is dominated by neighboring regulatory protein interactions along the thin filament, while cross-bridge binding is critical for maintaining or stabilizing thin filament activation as force develops. Moreover, we reveal that variations in filament and cross-bridge stiffness can alter Ca^2+^-sensitivity and cooperativity of skeletal muscle force production. In conclusion, these simulations show that multiple cooperative mechanisms combine to produce physiological force responses measured from muscle cells.

## Introduction

Striated muscle contraction is a Ca^2+^ dependent process. Ca^2+^ binding to troponin initiates thin filament activation, defined as exposure of sites along F-actin to which myosin can bind and form a cross-bridge (XB). In turn, XB binding can promote additional thin filament activation [1], [2]. The increase in force production with increasing [Ca^2+^] is highly non-linear, suggesting there is coupling between Ca^2+^-dependent and XB-dependent processes to augment thin filament activation and force production. The highly structured organization of the myofilament lattice (Figure S1) has led many investigators to suspect a role for spatial interactions between neighboring thin filament regulatory units (1 RU = 7 actin monomers+1 troponin complex+1 tropomyosin molecule) and/or neighboring XBs along the myofilaments to cooperatively augment thin filament activation [3]–[20]. Experiments have identified some possible forms of cooperativity between RUs along thin filaments and from XBs binding to actin. However a detailed picture of the Ca^2+^-dependent and XB-dependent cooperative mechanisms remains unclear because multiple cooperative processes are almost certainly coupled as muscle fibers contract.

**Figure 1:**
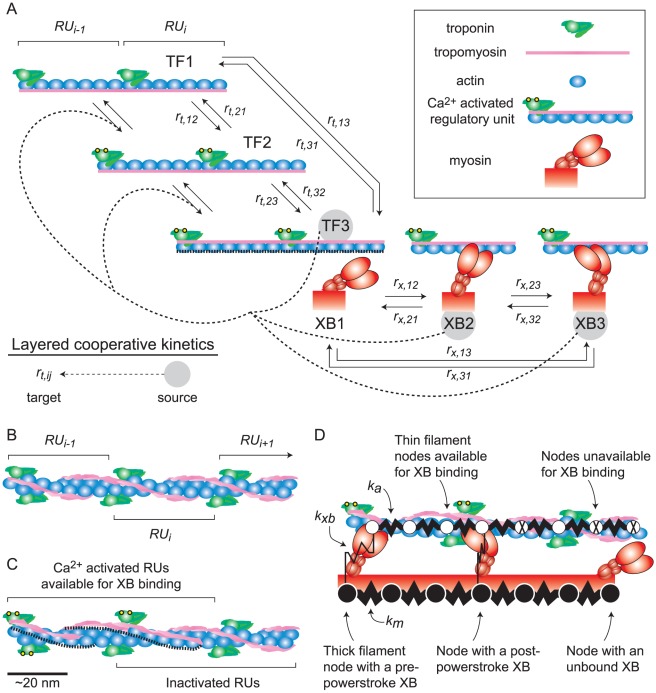
Kinetic forms of cooperativity are layered upon basic model kinetics. (A) Basic model kinetics combine two three-state cycles describing thin filament regulatory unit (RU) activation and cross-bridge (XB) binding [27]. Thin filament transition rates (*r_t,ij_*) between states TF1–TF3 represent spatial and kinetic behaviors consistent with troponin binding Ca^2+^ and tropomyosin movement to activate RUs, which allows myosin binding to actin. Dashed lines between TF2 and TF3, illustrate exposure of available binding sites along the thin filament. XB transition rates (*r_x,ij_*) are strain dependent between states XB1–XB3, representing transitions between unbound, bound pre-power stroke, and bound post-power stroke states. Cooperative pathways (dashed arrows) are layered upon basic model kinetics, amplifying thin filament activation kinetics at neighboring RUs. These layered, kinetic forms of cooperativity represent potential pathways where activation (TF3) or XB binding (XB2 or XB3) can act as a source of cooperativity to augment RU activation kinetics at neighboring RUs via targets *r_t,12_* or *r_t,23_*. (B) Adjacent RUs run along each of the helices that compose the thin filament. Thus, adjacent RUs along one helix face opposing thick filaments every ∼37 nm. Consistently, adjacent co-linear interactions between a thick and thin filament pair occur on alternate helices of the thin filament. (C) Therefore, certain regions of the thin filament can have RUs activated on both helices, either of the two helices, or neither of the two helices. (D) Similar to a finite element models, mechanics are simulated using a network of linear springs where forces balance at thick and thin filament nodes each time-step. The mechanical network comprises tunable spring constants *k_m_*, *k_a_*, and *k_xb_*, representing thick filament, thin filament, and myosin XB stiffness, respectively. Potential interactions for a co-linear thick and thin filament pair depict available thin filament nodes as shown in panel C. Those XBs extending from thick filament nodes occupy the co-linear plane, while XBs extending from neighboring thick filament nodes (not shown) lie outside of this plane.

Recent computational efforts have identified several potential mechanisms of cooperativity [21]–[27]. To study how these mechanisms rely upon the spatial and mechanical framework of the contractile filament lattice, we recently developed a spatially-explicit model that included Ca^2+^ regulation of individual RUs along thin filaments [27]. This modeling paradigm demonstrated a mechanical form of cooperativity that arises from compliant thick and thin filaments: XB binding to actin results in realignment between myosin heads and binding sites along the thin filament, which leads to additional XB recruitment as force develops (XB-XB cooperativity) [23], [25], [27]. However, our previous models did not account for kinetic properties of thin filament RU activation being influenced by XB binding (XB-RU cooperativity) or the activation state of neighboring RUs (RU-RU cooperativity). In this study we developed computational algorithms that allow thin filament RU activation rates to vary throughout a simulation, depending upon the spatial and biochemical states of neighboring RUs and XBs (Figure 1). This approach permitted a systematic investigation of potential cooperative mechanisms that, individually or in combination, influence Ca^2+^-sensitive force production in skeletal muscle. We simulate measurements of cooperative force production and illustrate relative contributions from spatial, kinetic, and mechanical characteristics of the half-sarcomere that determine cooperative activation of the thin filament in skeletal muscle.

## Results

### Ca^2+^ binding to troponin activates a thin filament distance spanning 9 to 11 actins

**Figure 2:**
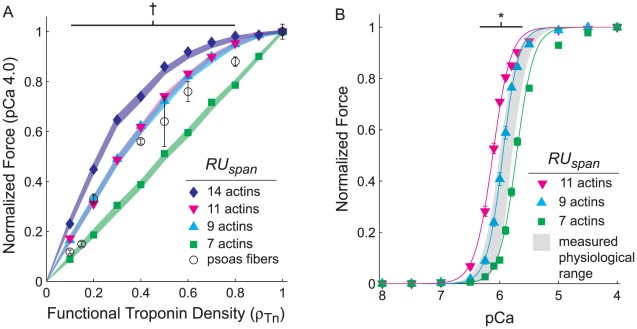
Regulatory unit activation span affects steady-state force production. Simulations where Ca^2+^ activated thin filament distance (*RU_span_*) varied while co-varying the fraction of troponin complexes capable of binding Ca^2+^ (*ρ_Tn_*) show maximal, steady-state force as a function of *ρ_Tn_* (A). Measurements from demembranated rabbit psoas muscle fibers (○ in panel A) show that maximal steady-state force increased with the fraction of troponin capable of binding Ca^2+^, replotted from Figure 4 of Regnier et al. [4]. *RU_span_* also affects the cooperative, steady-state force-pCa relationship (B). All values were normalized to the pCa 4.0 value within each simulation set and symbols represent mean±SE for measured and simulated data, where error bars lay within the symbol when not visible. These simulations combined all possible forms of cooperativity from sources TF3, XB2, or XB3, targeting *r_t,12_* and/or *r_t,23_* when applicable. Shaded underlays represent 95% confidence intervals for maximal force from 3-parameter Hill fits. † denotes where data from *RU_span_* values of 7 and 14 actins differed from *RU_span_* values of 9 and 11 actins (*p<0.05*). * denotes where data from all *RU_span_* values differed (*p<0.05*).

Because thin filament RUs are linked end-to-end via tropomyosin head-to-tail overlap, Ca^2+^ binding to a troponin and subsequent tropomyosin movement may activate more than 7 actins within a structural RU. We [4] and others [28] estimated this thin filament activation span to be 10–12 actins for skeletal muscle by using experimental approaches to titrate the number of functional troponin complexes along the length of thin filaments. If this Ca^2+^ activation span is correct, this would make Ca^2+^ binding to troponin capable of partially activating a region of the neighboring structural RUs. To simulate these experimental findings we co-varied model parameters *RU_span_* and *ρ_Tn_*, which control the length of RU activation along a thin filament (Table 1) and the functional troponin density, respectively. At the [Ca^2+^] that yields maximal steady-state behavior (pCa 4.0) with *ρ_Tn_* = 1, force and fractional thin filament activation were 973±14 pN and 0.993±0.004, consistent with our previous results [27]. These maximal values were not significantly different as *RU_span_* varied from 7–14 actins, suggesting complete activation of thin filaments can occur at pCa 4.0. As *ρ_Tn_* was increased from 0 to 1 at pCa 4.0, steady-state force increased linearly with *ρ_Tn_* when *RU_span_* was 7 actins, but became increasingly convex as *RU_span_* increased to 9, 11, and 14 actins (Figure 2A). Although a *RU_span_* of 14 actins produced the greatest non-linear increases in force as *ρ_Tn_* increased, *RU_span_* values of 9 and 11 actins predicted behaviors most consistent with skeletal muscle force measurements from our laboratory [4].

**Table 1:** Values of *RU_span_* compared to physiological thin filament structures.

*RUspan* (nm)	Structural RU	Actin monomers
37	1	7
50	1.33	∼9
62	1.66	∼11
75	2	14

As *RU_span_* increased from 7 to 9 to 11 actins, the Ca^2+^ sensitivity of the force-pCa relationship (*pCa_50_*) progressively increased by roughly 0.2 pCa units with little change in cooperativity (*n_H_*) (Figure 2B and Table 2), for simulations implementing all kinetic and mechanical forms of cooperativity with parameter values: *ρ_Tn_* = 1, *k_xb_* = 3 pN nm^−1^, *k_fil_* = 1X. The model predicted similar relationships for fractional thin filament activation versus pCa (data not shown), with slightly lower *pCa_50_* and *n_H_* values for thin filament activation level than force (Table 2). In combination, these results suggest a *RU_span_* of 14 actins produces supra-physiological activation and contractile responses, but a *RU_span_* value of 9 actins best describes skeletal muscle measurements from our laboratory [4]–[8]. Therefore, we used a *RU_span_* of 9 actins for all further simulations.

**Table 2:** Hill-fit parameters to steady-state force and thin filament activation responses versus pCa.

	Normalized Force		Fraction Available	
*RUspan* (actin monomers)	*pCa_50_*	*n_H_*	*pCa_50_*	*n_H_*
7	5.726±0.004	2.95±0.07	5.689±0.003	2.77±0.05
9	5.949±0.003	3.22±0.08	5.896±0.003	2.52±0.05
11	6.119±0.003	2.82±0.06	6.057±0.004	2.26±0.04
14	6.101±0.004	2.50±0.06	6.030±0.004	2.15±0.05

Hill parameter values are listed as mean±SD.

### Effects of different sources of cooperativity on the force-pCa relationship

**Figure 3:**
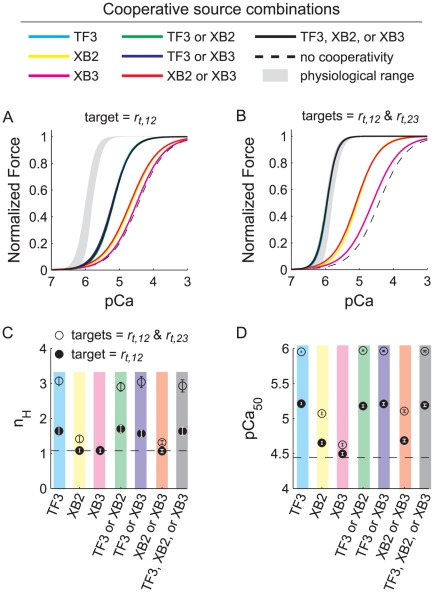
Multiple forms of cooperativity combine to simulate the physiological force-pCa relationship. Various combinations of cooperative thin filament activation kinetics affect the steady-state force-pCa response differently, shown for all possible source combinations when *r_t,12_* was targeted (A) versus both *r_t,12_* and *r_t,23_* being targeted in combination (B). Each line depicts the 3-parameter Hill fit to the simulated force-pCa response, while symbols show *n_H_* (C) and *pCa_50_* (D) values for these fits, with error bars representing 95% confidence intervals. All simulations used standard parameter values: *ρ_Tn_* = 1, *k_xb_* = 3 pN nm^−1^, *k_fil_* = *k_m_* = *k_a_* = 1X, and a *RU_span_* of 9 actins. The dashed lines illustrate an example simulation in the absence of cooperative thin filament activation kinetics, which compares well with prior studies [25], [27], [43] after adjusting for *K′_1_* (Table 3). The shaded underlay in panels A and B represents the measured physiological range from Figure 2.

To investigate the effect of individual versus combined mechanisms of cooperativity on Ca^2+^-sensitivity and cooperativity of force production, we systematically assessed the influence of each kinetic form of cooperative thin filament activation (*i.e.* all possible combinations of source-target cooperativity illustrated in Figure 1A and further described the Materials & Methods). All simulations (Figure 3) used standard parameter values fixed at *RU_span_* = 9 actins, *k_xb_* = 3 pN nm^−1^, and *k_fil_* = 1X. Neighboring activated RUs (TF3 as the source of RU-RU cooperativity) provided the greatest influence on the force-pCa relationship, followed by low-force XBs at neighboring RUs (XB2 as the source of XB-RU cooperativity), and finally high-force XBs at neighboring RUs (XB3 as the source of XB-RU cooperativity). This demonstrates a hierarchy of influence on the force-pCa relationship for the three kinetic sources of cooperativity: TF3>XB2>XB3. Throughout a simulation there are more RUs activated than there are XBs bound, which likely promotes this hierarchy. In addition, the finding that low-force bearing XBs (XB2 in Figure 1A) may contribute more to cooperative thin filament activation than high-force bearing XBs (XB3 in Figure 1A) is an intriguing prediction that supports a role for low-force (weak binding) XBs in the activation process [29], [30] as well as the idea of a Ca^2+^-dependent equilibrium between low-force and high-force XBs in modulating thin filament activation [3], [5], [10], [11], [13].

There is also a hierarchy of influence for the thin filament transition rate(s) being targeted by a cooperative mechanism. Targeting *r_t,12_* or *r_t,23_* in combination (Figure 3B and S2G–I) produced a greater cooperative response than *r_t,12_* alone (Figure 3A and S2A–C), both which produced greater responses than *r_t,23_* alone (Figure S2D–F). This demonstrates a synergistic effect of targeting *r_t,12_* and *r_t,23_* in combination that was consistent across all simulations, even for the least influential source of cooperativity (XB3). The results suggest XB binding may play an important role in preventing tropomyosin moving back to an inhibitory position, stabilizing RU activation and augmenting thin filament activation throughout the half-sarcomere. This set of simulations supports the hypothesis that RU-RU cooperativity is the dominant source of cooperativity in skeletal muscle, and could influence thin filament transition rates that are downstream from Ca^2+^ binding of troponin C, such as the troponin C-troponin I interaction, to facilitate greater tropomyosin mobility.

### Effects of XB binding on the magnitude and rate of thin filament activation

**Figure 4:**
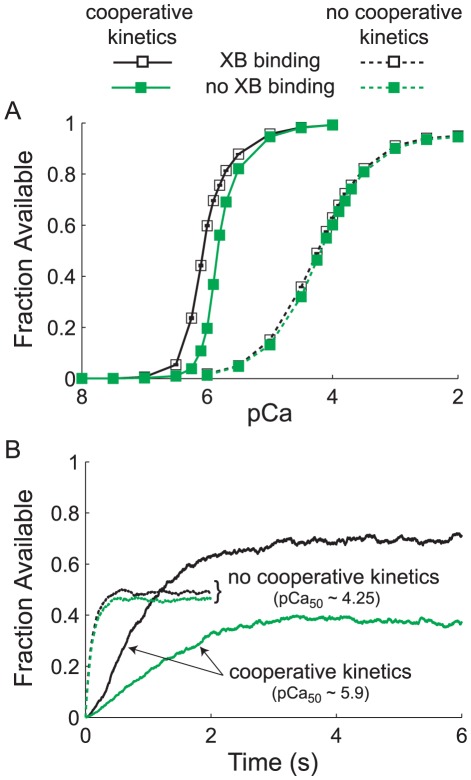
Cross-bridge (XB) binding stabilizes and augments thin filament activation. (A) Steady-state thin filament activation is plotted against pCa for simulations where standard parameter values were applied (see Figure 3), in the presence and absence of kinetic forms of cooperativity. XBs produce larger increases in fractional thin filament activation when cooperative kinetics were present, evidenced by the increase in activation in the presence versus absence of XB binding. These XB-dependent increase in thin filament activation are greatest at submaximal pCa levels, shown by the differences between the two curves when kinetic forms of cooperativity were implemented. Symbols represent mean±SE, where error bars reside within the symbol when not visible. (B) Average thin filament activation is plotted against time for simulations near the *pCa_50_* value of the steady-state force-pCa response (Figure 3), with (pCa = 5.9) and without (pCa 4.25) kinetic forms of cooperativity. These time series activation traces demonstrate the representative slowing of thin filament activation kinetics and increased magnitude of thin filament activation due to XBs in the presence of cooperativity. All simulations used standard parameter values: *ρ_Tn_* = 1, *k_xb_* = 3 pN nm^−1^, *k_fil_* = *k_m_* = *k_a_* = 1X, and *RU_span_* = 9 actins.

To isolate the effects of XB binding from other sources of cooperativity on thin filament activation we investigated the fractional activation of thin filaments by Ca^2+^, in the presence and absence of XB binding. XB binding provided the greatest increases in thin filament activation at submaximal pCa values (Figure 4A) when all possible mechanisms (kinetic and mechanical) of cooperativity were implemented. In contrast, in the absence of kinetic forms of cooperativity, XB binding had a minimal effect on thin filament activation across the entire pCa range (Figure 4A). Moreover, the activation and force traces shown in Figure 4B illustrate that including kinetic forms of cooperative thin filament activation dramatically slows the rate of thin filament activation (*k_TF,act_*). The full *k_TF,act_*-pCa relationships are shown in Figure S3. These simulations (Figure 4B) also demonstrate a significant XB-dependent increase in the magnitude of thin filament activation when kinetic forms of cooperativity were implemented, consistent with the steady-state results shown in Figure 4A.

### Mechanical properties of the filaments and cross-bridges influence cooperative force production

**Figure 5:**
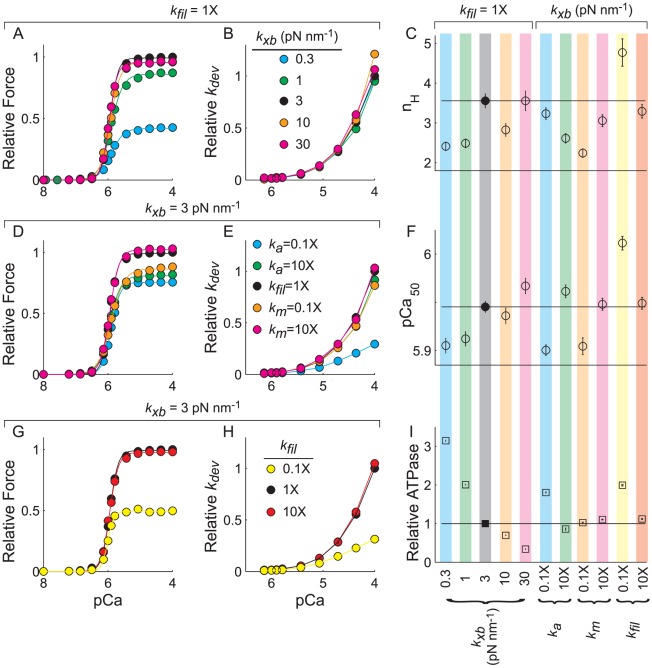
Mechanical properties of the myofilament lattice influence cooperative force production and maximal XB turnover. Simulation results for steady-state magnitude and rate of force development (*k_dev_*) are plotted against pCa as (A–B) XB stiffness (*k_xb_*) varied, (D–E) thick or thin filament stiffness (*k_m_* or *k_a_*) varied independently, or (G–H) both filament stiffness values (*k_fil_*) varied simultaneously. Parameter values from 3 parameter Hill fits to these force-pCa relationships demonstrate that mechanical properties (*i.e.* stiffness) of they myofilament lattice can influence cooperativity (*n_H_*; panel C) and Ca^2+^ sensitivity (*pCa_50_*; panel F) of force production. The rates of XB cycling or turnover directly correlate with ATPase values (I). All relative values were normalized to results for the standard parameter values (solid black circle) of *k_xb_* = 3 pN nm^−1^, *k_fil_* = *k_m_* = *k_a_* = 1X, *RU_span_* = 9 actins, and all possible forms of cooperativity were implemented for all simulations. Symbols represent mean±SE in all panels except C and F, and error bars reside within the symbol when not visible. Symbols in panels C and D depict predicted parameter values with error bars representing 95% confidence intervals.

To investigate how the mechanical properties of XBs and the myofilaments influence cooperative force production, rate of force development (*k_dev_*), or rate of XB turnover (ATPase), we varied XB, thick filament, and thin filament spring constants (*k_xb_*, *k_m_*, and *k_a_*, respectively). Standard filament stiffness values (*k_xb_* = 3 pN nm^−1^; *k_fil_* = *k_m_* = *k_a_* = 1X) resulted in maximal force and *k_dev_* values of 973±14 pN and 32.6±0.1 s^−1^, consistent with results shown in Figure 2. Decreasing *k_xb_* decreased maximal force and *k_dev_* (Figure 5A–B), diminished cooperativity (Figure 5C) and Ca^2+^ sensitivity (Figure 5F) of the force-pCa relationship, and elevated XB cycling (Figure 5I). Increasing *k_xb_*, however, produced more heterogeneous dynamics. A *k_xb_* of 10 pN nm^−1^ increased maximal *k_dev_* by 20%, but resulted in minimal shifts in the force-pCa relationship and a small decrease in *n_H_*. Further increasing *k_xb_* to 30 pN nm^−1^ increased maximal *k_dev_* by 6%, but produced a small ‘left-shift’ in the force-pCa relationship, slightly increasing *pCa_50_* to 5.96. For all simulations that varied *k_xb_*, stiffer XBs led to slower rates of XB turnover (Figure 5I) due to decreased ability of myosin to diffuse to a binding site. While these changes in maximal force production and ATPase are consistent with our previous observations [27], the findings that XB stiffness can influence the cooperative nature of the force-pCa relationship and *k_dev_* reveals a new role for mechanics of filaments and XBs in cooperative binding processes.

With *k_xb_* fixed at 3 pN nm^−1^, decreasing thin filament stiffness (*k_a_*) 10-fold decreased maximal force by 25% (Figure 5D), slowed *k_dev_* by 70% (Figure 5E), and reduced *pCa_50_* by ∼0.5 pCa unit (Figure 5F). Conversely, increasing *k_a_* 10-fold decreased maximal force by 20%and slightly increased *pCa_50_*, but similarly slowed *k_dev_*, albeit by only 9%. Comparable decreases in thick filament stiffness (*k_m_*) reduced maximal force by 12%, slowed *k_dev_* by 14%, and reduced *pCa_50_*, while a 10-fold increase in *k_m_* produced relatively minimal changes in the force-pCa response. Thus, these results suggest changes in thin filament stiffness (relative to thick filament stiffness) have a larger influence of on Ca^2+^ mediated activation and force development.

However, there is a coupling mechanism that arises from the relative stiffness values between the thin and thick filaments, as simultaneously reducing both *k_a_* and *k_m_* 10-fold (*k_fil_* = 0.1X) resulted in a 50% reduction in maximal force (Figure 5G), a relatively large increase in *n_H_* and *pCa_50_* compared to the other simulations (Figure 5C, F), a 70% reduction in *k_dev_* (Figure 5H), and a doubling in the XB turnover rate (Figure 5I). In contrast, simultaneously increasing both *k_a_* and *k_m_* 10-fold (*k_fil_* = 10X) produced minimal shifts in the force-pCa relationship (Figure 5G) or XB turnover rate, and a minor (5%) increase in maximal *k_dev_* (Figure 5H). Even though *k_xb_* was fixed throughout these simulations, shifts in the force-pCa relationship arise from a redistribution of the bound XB populations throughout a simulation. This illustrates the influence of mechanical forms of cooperativity due to strained compliant filaments as force develops throughout a simulation, causing realignment of binding sites along the thin filament with respect to XB locations along the thick filaments. This realignment leads to additional XB recruitment (*i.e.* XB-XB cooperativity) and affects the force-pCa relationship when combined with kinetic forms of cooperative thin filament activation. Importantly, these stiffness-dependent shifts in *k_dev_* and *pCa_50_* were not seen in previous analyses [27].

In summary, decreases in XB or myofilament stiffness increase XB-XB cooperativity, increasing rates of XB turnover, but diminishing force, *n_H_*, *pCa_50_*, and *k_dev_*. These results support our previous simulations [27], illustrating the counter-intuitive influence of XB-XB cooperativity on the dynamics of force development, where a more compliant filament lattice reduces transmission of force production and slows the apparent rate of force development even though there is increased XB recruitment and turnover. These simulations predict a fascinating relationship between mechanical and kinetic forms of cooperativity that are coupled to regulate cooperative thin filament activation and force development in a muscle fiber.

## Discussion

This study reveals how multiple cooperative processes (alone and in combination) affect force production in skeletal muscle by simulating contractile and regulatory protein dynamics within a spatially-explicit, multi-filament model of the half-sarcomere. Both our empirical measurements and model predictions show that a single troponin complex regulates a span of 9–11 actin binding sites along skeletal muscle thin filaments. We also show that variations in effective myofilament lattice stiffness can influence cooperativity (*n_H_*) and Ca^2+^-sensitivity (*pCa_50_*) of the force-pCa relationship. These simulations illustrate how kinetic and mechanical forms of cooperativity combine to control the magnitude and rate of force production during skeletal muscle contraction.

### Spatial mechanisms of cooperativity that regulate thin filament activation

In previous experiments we reduced cooperative interactions between neighboring RUs along thin filaments in demembranated rabbit psoas muscle fibers by extracting native troponin C (TnC), then reconstituting troponin complexes with varying mixtures of native TnC and a mutant TnC (D27A, D63A) that cannot bind Ca^2+^ at N-terminal sites I and II [4]. Those results showed that reduced spatial coordination between neighboring RUs along the thin filament can limit force production. By varying the number of actins that become available for myosin binding upon Ca^2+^ activation of a RU *in silico* (via the model parameter *RU_span_*), simulation results agreed best with our prior measurements when the *RU_span_* was set at 9–11 actins (Figure 2). Simulations also predict that Ca^2+^ binding to troponin is unlikely to activate a thin filament span greater than two structural RUs (∼14 actin monomers), because this leads to a hypersensitive force response. An estimated *RU_span_* of 9–11 actins agrees well with other studies [17], [19] and our previous estimate of 10–12 actins [4], although shorter than estimates from single molecule [31] or early muscle fiber [14] studies. Therefore, our empirical and computational results suggest that the functional activation span of a RU is greater than the 7 actin monomers of a structural RU, but remains bracketed by the pair of neighboring troponin molecules surrounding a troponin complex. Thus coupling between neighboring RUs along the thin filament can influence spatial and kinetic processes of activation, albeit at relatively local regions along the thin filament.

### Kinetic sources of cooperative thin filament activation

The current model allowed us to explore the relative influence on thin filament activation of various mechanisms, such as the number of proximal activated RUs or bound XBs that augment activation kinetics at neighboring RU (Figure 1A). We find that adjacent activated RUs (state TF3; Figure 1) provided the greatest source of cooperative thin filament activation, with RU-RU cooperativity producing the largest increases in *pCa_50_* and *n_H_* of the force-pCa relationship. The next most effective contributors to cooperative thin filament activation were neighboring, bound XBs: states XB2 and XB3, respectively. In addition, combining multiple forms of RU-RU and XB-RU cooperativity that jointly targeted multiple thin filament activation rates (*r_t,12_* and *r_t,23_*, Figure 1) influenced the force-pCa relationship more greatly than individual forms of kinetic cooperativity. These findings suggest that the physiological force-pCa relationship arises from multiple, cooperative processes at the molecular level that coordinate thin filament activation and XB binding.

The cooperative force-pCa relationship may be more sensitive to RU activation than XB binding because the fractional pool of activated RUs is always greater than the fractional pool of bound XBs (∼100% vs. ∼15% at pCa 4.0). Within this 15%, roughly 2/3 of the bound XB population resides in the low-force bearing state throughout a simulation, which may explain the why low-force XBs (XB2) contribute more greatly to cooperative activation than high-force XBs (XB3). Therefore, the relative sensitivity to multiple sources of cooperativity may vary as RU activation and XB kinetics vary with fiber type and taxa.

### Mechanical sources of cooperative force development

The spring constants of the XBs and the myofilaments are important determinants of the force-pCa relationship and the rate of force development (*k_dev_*). Compliance in the filament lattice leads to a spatial redistribution of binding sites in response to local XB force generation. That redistribution, in turn, may increase recruitment of additional XBs and may influence RU activation. These varied cooperative dynamics would not occur within a system of inextensible filaments, because there would be no heterogeneity in the transmission of forces throughout the filaments as occurs herein with varied values for XB, thin- or thick-filament stiffness (*k_xb_*, *k_a_* or *k_m_*, respectively). Interestingly, *isolated* decreases in *k_xb_*, *k_a_* or *k_m_* generally reduce maximal force, *pCa_50_*, and *n_H_*, indicating a diminished cooperative force response. In contrast, *simultaneous* decreases in *k_a_* and *k_m_* (*k_fil_* = 0.1X) increased *n_H_* and *pCa_50_*, indicating a more cooperative force-pCa relationship. Thus, the relative influence of multiple forms of cooperativity depends upon the relative stiffness difference between thick and thin filaments, where greater divergence between thick and thin filament stiffness values or increased XB flexibility diminishes the potency with which cooperative mechanisms augment force development.

We note that the thick versus thin filaments stiffness cannot deviate too much in their relative stiffness values, otherwise nearly all of the realignment will reside in the more compliant set of filaments, which reduces the capacity for cooperative force production. Because there are twice as many thin filaments as thick filaments, variations in *k_a_* alone affected the cooperative force response more than comparable changes in *k_m_*, alone, making effective variations in myofilament lattice stiffness more sensitive to *k_a_* than *k_m_*. In contrast, simultaneous decreases in *k_a_* and *k_m_* reduce the relative stiffness difference between the filaments, allowing them to undergo comparable levels of compliant realignment to facilitate mechanical and kinetic forms of cooperative force production at mid-pCa levels, even though maximal force values at pCa 4.0 may be significantly compromised. This implies that the most efficient levels of cooperative force production may arise from relatively stiff thick and thin filament values, at the same order of magnitude, consistent with physiological observations in vertebrates [32], [33].

### Comparisons with previous cooperative models of muscle contraction

Our spatially-explicit models provide the unique ability to explore how spatial, kinetic, and mechanical characteristics of thin filament activation and XB binding throughout the half-sarcomere influence cooperative activation. As summarized below, our findings are consistent with results from previous studies [24], [34]–[37], which continue to suggest that multiple cooperative mechanisms are almost always required, in combination, to simulate physiological measurements of cooperative force production.

Consistent with our observations, RU-RU cooperativity has been the most potent form of cooperative thin filament activation in previous computational studies [24], [34]–[36]. However, XB binding consistently contributes a synergistic role that maintains and augments thin filament activation to recruit additional XBs as force develops [34], [35]. The kinetic forms of cooperativity significantly slow the apparent rate of activation and force development (*k_TF,act_*, *k_dev_*, or *k_tr_*, depending upon the kinetic parameter in question) because these cooperative mechanisms increase the pool of activated RUs and bound XBs as force develops over time [22], [26]. Therefore, the kinetic transition rates underlying thin filament activation and XB cycling may differ greatly from the apparent rates of cooperative force development and relaxation throughout a simulation or a muscle contraction [22], [34], [35].

Recently, Geeves et al. [37] combined solution kinetic measurements of myosin binding to regulated thin filaments and a continuous flexible chain model of RU activation, rather than assuming a rigid *RU_span_* value as modeled here and elsewhere [27], [35]. Their measurements suggest that strong XB binding can cooperatively activate RUs along the thin filament, even though the rate of myosin binding is regulated by the position of tropomyosin along the thin filament. Consistent with our model of thin filament activation, their results illustrate that the rate of myosin binding to actin may be limiting force production, rather than the rate of RU activation because dynamic movement of tropomyosin is more rapid than troponin I detachment from actin. Predictions from their continuous flexible chain model also suggest Ca^2+^-binding or XB binding may lead to ‘clusters’ of force bearing XBs along the thin filament (over a length of 25–50 nm), particularly near the onset of contraction at low [Ca^2+^]. This distance agrees well with our estimates of *RU_span_* and supports the idea that cooperative activation occurs at relatively local regions along the thin filament, consistent with clustered islands of XB binding throughout the half-sarcomere that have been demonstrated by previous spatially-explicit models [23], [26].

### Conclusions

These simulations show that RU-RU cooperativity occurs rapidly and dominates filament activation early in the contractile process, while the influence of XB-RU and XB-XB cooperativity occurs more slowly, becoming increasingly important as force continues to develop. Moreover, the mechanical characteristics of the XBs and the myofilaments greatly influence these mechanisms. The relative speed and influence of these various cooperative mechanisms favors the interpretation that rapid and complete activation of skeletal muscle thin filaments leads to maximal force production in a fiber, thereby allowing graduated recruitment of motor units to dictate contractility of the whole muscle. In contrast, every muscle cell in the heart contracts during each heartbeat, which may require a redistribution in the hierarchy of influence from multiple kinetic and mechanical forms of cooperativity. For instance, the relative contribution of XB-RU cooperativity may increase in cardiac muscle to provide for more ‘local’ regulation of force development within a RU as XB binding events enhance Ca^2+^ binding to troponin or maintain RU activation. Consistently, the dominant influence of RU-RU cooperativity may diminish, as the *RU_span_* in cardiac muscle appears to be less than the 7 actins of a structural RU [28], [38]. As discussed within previous computational studies, cardiac muscle may also involve a ‘negative’ or ‘anti’ cooperativity that favors rapid thin filament deactivation to locally control contraction throughout each heartbeat [34]–[36]. The computational methods developed herein provide unique tools for examining and discerning kinetic and mechanical differences between skeletal and cardiac muscle contraction at the molecular level, and importantly, how regulation of contraction may be altered with damage or disease.

## Materials and Methods

We build upon our previous spatially-explicit model of muscle contraction that simulated Ca^2+^-regulated XB interactions in a half sarcomere consisting of 4 thick filaments and 8 thin filaments, where state transitions were modeled with Monte Carlo methods [27]. As described below, we now add mechanisms of cooperativity to the model that influence thin filament activation kinetics as the biochemical state of neighboring thin filament regulatory units (RU) or cross-bridges (XB) vary throughout the myofilament lattice (Figure 1A).

### Model mechanics

Similar to a finite element model, Ca^2+^-activated thin filament regulatory processes and thick-to-thin filament XB interactions are simulated within a network of linearly elastic springs. Within this network, forces and deformations occur along the axial direction of the filaments (Figure 1D), providing a linear system of equations that represents a one dimensional instantaneous force balance throughout the half-sarcomere (Eq. 1). Individual thick or thin filaments consist of 61 or 91 elastic spring elements, respectively, linked end-to-end at ‘nodes’ about which forces balance (60 thick filament nodes and 90 thin filament nodes). Similar to our previous simulations [23], [25], [27], thick and thin filament spring constants are *k_m_* = 6060 and *k_a_* = 5230 pN nm^−1^ for resting (unstrained) elements of length 14.3 and 12.3 nm, respectively, which constitute half-sarcomere long thick and thin filaments of ∼860 ( = 60×14.3 nm) and ∼1110 ( = 90×12.3 nm) nm. Node locations coincide with model structures that represent myosin XBs along thick filaments and actin monomers along thin filaments. Stoichiometrically this leads to 6 myosins every ∼43 nm of thick filament that are co-linearly aligned with, and may bind to, 3 actin monomers every ∼37 nm of thin filament [27]. Because different ratios and arrangements of the thick and thin filaments can lead to different levels of XB recruitment and turnover [27], it is plausible that different model geometries or varied stoichiometry could influence kinetic and mechanical forms of cooperativity investigated in this study.

As further discussed below, Ca^2+^ regulation of contraction stems from a sub-set of thin filament nodes that are co-located with model structures representing troponin. The XB spring constant (*k_xb_*) was primarily fixed at 3 pN nm^−1^ to be consistent with parameter ranges used in previous simulations and recent estimates from cellular experiments [39]–[41]. Collapsing this geometry into a matrix of spring constants (***K***), and a vector of boundary conditions (***V***) allows us to solve the instantaneous balance of forces within the elastic network to determine a vector of unknown node locations (***P***) given the state of all XBs [23], [25]–[27]:
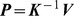
(1)We assume that inertial and viscous interactions are negligible under isometric conditions [42]. Some simulations scaled the value of *k_m_*, *k_a_*, or *k_xb_* independently, while other simulations simultaneously scaled the values of *k_m_* and *k_a_*. The scalar multiple affecting individual filament stiffness values precedes X, such as *k_m_* = 10X or *k_a_* = 10X to represent either *k_m_* or *k_a_* becoming 10 times stiffer. Simulations where both *k_m_* and *k_a_* varied simultaneously are listed as *k_fil_* = 0.1X, for example, if both thick and thin filament stiffness values decreased 10 fold.

### Spatial determinants of cooperative thin filament activation

Compared to our previous model [27], the current model has an additional parameter representing the fraction of functional troponin molecules along thin filaments (*ρ_Tn_*, *i.e.* the density of troponin capable of binding Ca^2+^). Throughout any single simulation *ρ_Tn_* is set at the beginning of each simulation via Monte Carlo algorithms that randomly ‘knocked out’ troponin complexes along each thin filament. *RU_span_* is another new model parameter, representing the length of thin filament near a troponin molecule that becomes available for myosin binding upon Ca^2+^ activation of a RU (Figure 1B–D). Because thin filaments are modeled as a discrete set of thin filament nodes or ‘actin binding sites’ along thin filaments, *RU_span_* effectively takes on discrete values (Table 1). Thus, *ρ_Tn_* and *RU_span_* collectively establish the total number of actin nodes available to bind myosin XBs, simulating Ca^2+^-regulation by troponin and tropomyosin. The kinetic state at a troponin site is applied to actin nodes within the distance of *RU_span_*. Therefore, when *RU_span_* assumes the distance of a structural RU or 7 actins, each troponin will control the state of all actin nodes within a single RU. However, as *RU_span_* increases, there become regions for overlap where a single thin filament node may be influenced by multiple, adjacent troponins along one of the two helices making up a thin filament. In these cases, we apply the most activated state (*i.e.* TF3>TF2>TF1) between the two influential troponin molecules to represent the state of the thin filament node in question. This spatially-explicit thin filament activation algorithm differs from our prior models [27], [43] that effectively assumed a *RU_span_* of 1 structural RU or 7 actins, which dictates no possible overlap between the ‘spatial regions of influence’ among adjacent RUs along the thin filament.

### Basic model kinetics

Thin filament activation and XB kinetics are controlled through two coupled, three-state cycles (Figure 1A), similar to our previous model [27]. Thin filament states represent troponin without Ca^2+^ bound (TF1), Ca^2+^ bound to troponin (TF2), and tropomyosin movement to a position permitting myosin binding with actin (TF3). Thin filament states TF1 and TF2 represent inactivated RUs where myosin cannot bind with actin. XB states are unbound (XB1), bound pre-power stroke (XB2), or bound post-power stroke (XB3). XB1 represents an unbound state that does not bear force. The bound states represent low-force (XB2) and high-force (XB3) bearing conformations (although the specific force borne by any XB depends upon XB strain and stiffness).

**Figure 6:**
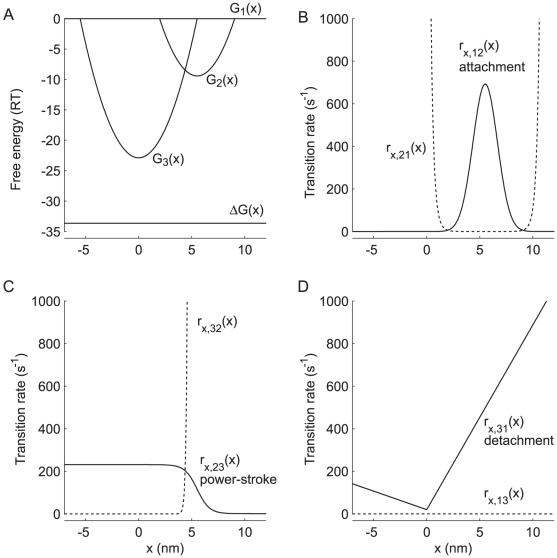
Position dependent XB kinetics for *k_xb_* = 3 pN nm^−1^. Position-dependent free energy differences (A) and transition rates (B–D) for the XB states illustrated in Figure 1A are plotted against, x, which represents the position difference between a particular pair of actin and myosin nodes supporting a bound XB. (A) The top horizontal line shows the free energy of the detached state (G_1_(x)), where the difference between the two horizontal lines represents the standard free energy drop over a full XB cycle (ΔG(x)). Each parabolic free energy well G_2_(x) and G_3_(x) represents bound states XB2 and XB3, respectively. For panels B–D, solid lines represent forward transition rates, and dashed lines represent reverse transition rates as formulated in Tanner et al. [27].

Model kinetics are stochastically driven with Monte Carlo algorithms by drawing a random number (*n*) from a uniform distribution over the open interval (0,1). Any single transition probability (*p_ij_*) from state *i* to state *j* depends upon the transition rate (*r_ij_*) and time-step (*dt* = 1 ms): *p_ij_* = *r_ij_dt*. Transition probabilities are calculated each time-step to determine whether the Markov process underlying behavior of each node undergoes a forward transition, reverse transition, or remains as is:
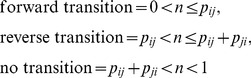
(2)Basic thin filament transition rates are listed in Table 3 and position-dependent XB transition rates are shown in Figure 6 for *k_xb_* = 3 pN/nm. A state transition will occur within a single time-step if *p_ij_*≥1, as would occur for basic model parameters *r_t,12_* or *r_t,13_* if [Ca^2+^] exceeded 2 mM (pCa≈1.7). If corresponding thick and thin filament nodes representing an attached XB become unfavorably aligned a number of XB transitions could take place within a single time-step (if *r_x,ij_(x)*>1000 s^−1^in Figure 6).

**Table 3:** Thin-filament transition rates (*ξ* = 100).

Equilibrium	Basic forward transition rate (*Ψ* = 1)	Cooperative forward transition rate (*Ψ* = 100)	Reverse transition rate (independent of *Ψ*)
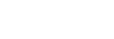 = 10^3^ (M^−1^)	*r_t,12_* = 5×10^4^ (M^−1^ s^−1^)	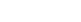 = 5×10^6^ (M^−1^ s^−1^)	*r_t,21_* = 50 (s^−1^)
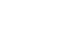 = 10	*r_t,23_* = 10 (s^−1^)	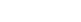 = 1000 (s^−1^)	*r_t,32_* = 10 (s^−1^)
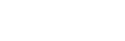 = 10^−4^ (M)	*r_t,31_* = 5 (s^−1^)	*r_t,31_*	*r_t,13_* = 5×10^4^ (M^−1^ s^−1^)

### Kinetic determinants of cooperative thin filament activation

Within any simulation where cooperative thin filament activation kinetics were implemented, thin filament transition rates varied with the state of neighboring thin filament or XB nodes within an adjacent RU (Figure 1A). This introduces kinetic forms of cooperativity where *r_t,12_* and *r_t,23_* take on one of two values: their basic value if neighboring RUs are in state TF1 or TF2 and/or neighboring actin nodes within an adjacent RU do not have a XB attached, or their cooperative value if neighboring RUs are activated to state TF3 and/or neighboring actin nodes within adjacent RUs have a XB attached (Table 3). This creates pairs of ‘source-target’ cooperativity (Figure 1A) where behavior within adjacent RUs becomes the source of the cooperativity (either states TF3, XB2, or XB2), which can augment thin filament activation of the RU in question (either *r_t,12_* and/or *r_t,23_* become the target). This approach permits the relative strength of these pathways to increase, allowing us to examine sources of cooperativity stemming from proximal, activated RUs or proximal bound XBs. These kinetic forms of cooperativity can be combined from any RU-RU or XB-RU cooperative pathway outlined by the dashed arrows in Figure 1A, where individual pathways use TF3, XB2, or XB3 as the sources that can target RU activation rates *r_t,12_* and/or *r_t,23_* together or in combination (which we simply refer to as the RU target). Therefore, individual pathways can be listed as: TF3-RU, XB2-RU, and XB3-RU. In addition, these individual pathways can be combined two-fold within a simulation, where TF3-RU or XB2-RU, TF3-RU or XB3-RU, and XB2-RU or XB3-RU become the three possibilities for coupled cooperative kinetics that may have a greater influence than either single pathway (Figure 3 and S2). Finally, these three individual pathways may also be combined within a simulation such that cooperative activation of the thin filament may follow from the combination of TF3-RU or XB2-RU or XB3-RU.

Thus, any single thin filament transition probability (*p_ij_*) takes one of two probabilities: i) either the basic probability: *p_ij_* = *r_t,ij_dt*; or ii) the cooperative probability that increases *p_ij_* by the scale factor (*Ψ* = 100) to reflect the cooperative value (Table 3). Again, a state transition will occur within a single time-step if *p_ij_*≥1, and would occur for the cooperative thin filament transition rate *r′_t,12_* if [Ca^2+^] exceeded 0.2 mM (pCa≈3.7) or *r′_t,23_*. Across all simulations we maintain identical scaling for any cumulative source-target combinations, forcing the relative weight of any kinetic cooperativity to be similar, independent of the various sources in combination (TF3, XB2, or XB3) that were queried within a particular simulation. This scaling approach enhanced our capacity for separating cooperative influences from different sources, and minimized variability in the numerical value applied to any cooperative thin filament activation rate between simulations.

As an example, envision simulations comparing XB3 alone versus XB2 or XB3 combined as sources of cooperativity. This scaling approach dictates that any differences between these two simulations will be attributed to the larger pool of proximal bound XBs for the combined case. The larger pool of bound bridges (when considering XB2 or XB3) increases overall thin filament activation compared to XB3 alone (Figure 3), although numerical increases in the probability of activation for any single transition remains similar for both simulations. In the event that neither of these cooperative conditions were met, *p_ij_* does not change from the basic, non-cooperative value.

The force-pCa relationship is highly sensitive to the rate of Ca^2+^ association and dissociation from troponin, which is represented by the values of *r_t,12_* and *r_t,21_* (Figure 1A and Table 3). Within our prior model [27], this equilibrium is given by *K_1_ = r_t,12_/r_t,21_* = 10^5^ M^−1^. With the addition of cooperative kinetics within this model, Ca^2+^ sensitivity of thin filament activation significantly increased in preliminary simulations (from≈pCa 6.0 to 8.0) when *r_t,12_* was scaled by *Ψ*. Thus, basic model kinetics were modified from our prior study [27] by rescaling *K_1_* by a second scale factor *ξ* ( = 100) to re-normalize *pCa_50_* values near 6.0. This scaling makes effective Ca^2+^ affinity of troponin during a cooperative simulation≈*K_1_^′^Ψ*, a product which largely dictates *pCa_50_* of the force-pCa relationship. As further discussed in Text S1 and shown in Figure S4, we ran a number of preliminary simulations over an extensive range of *Ψ* values and a near-exhaustive set of potential source-target combinations. Based on these findings we focused on a relatively small set of possible kinetic combinations of cooperativity that predicted behaviors consistent with physiological measurements from our laboratory [4]–[8].

### Data analysis

Identical to prior analysis [27], we used a 3-parameter Hill fit to describe the steady-state force, fraction of actin nodes available to bind with myosin (occupying state TF3), and actomyosin ATPase rate (with units of ATP myosin^−1^ s^−1^) as a function of pCa. These fits provide parameter estimates describing the maximal value as [Ca^2+^] approaches infinity (*X_max_*), the [Ca^2+^] producing half-maximal force (*pCa_50_*), and the slope at *pCa_50_* (*n_H_*). We also estimated the rate of force development (*k_dev_*) via the duration that any single force trace required to achieve half steady-state force (*t_1/2_*): *k_dev_* = ln(2)/*t_1/2_*. Similarly, the rate of thin filament activation (*k_TF,act_*) was calculated from the half-time to steady-state fractional thin filament activation. Statistical differences were assessed via a one-way ANOVA followed by a Tukey-Kramer multiple comparison of the means (*p*≤0.05). All simulations and analysis were performed using custom algorithms written in Matlab (The Mathworks, Natick, MA., USA).

## Supporting Information

Figure S1 Vertebrate striated muscle structure of a half-sarcomere. Computational algorithms of this spatially-explicit model represent thick filaments and thin filaments of half-sarcomere length from the M-line to the Z-line (A, color scheme consistent with Figure 1). These filaments are organized in the hexagonal lattice structure consistent with vertebrate striated muscle, more obviously demonstrated by a cross-sectional view of the A-band (B). Each filament also consists of a helical pitch describing myosin cross-bridges (XBs) extending from the thick filament or the intertwined filamentous actin helices, along which the thin filament regulatory proteins are located (C). Our computational representation of myofilament lattice organization accounts for these structural components of the muscle.
(PDF)

Figure S2 Multiple cooperative pathways combine to produce the physiological force-pCa relationship. Sensitivities of force and fractional thin filament activation are illustrated for all combinations of kinetic cooperativity. (A) Lines show 3-parameter Hill fits to steady-state force-pCa responses for *r_t,12_* being targeted independently, along with symbols depicting *n_H_* (B) and *pCa_50_* (C) values from fits to both force-pCa and fraction available-pCa relationships. Comparable simulation results are shown for *r_t,23_* being targeted independently (D–F), or *r_t,12_* and *r_t,23_* being targeted in combination (G–I). Dashed lines illustrate simulation results in the absence of cooperative kinetics. Error bars represent 95% confidence intervals on the fitted parameter values for all panels. All simulations used standard parameter values of *k_xb_* = 3 pN nm^−1^, *k_fil_* = *k_m_* = *k_a_* = 1X, and *RUspan* = 9 actins, combining all possible forms of cooperativity.
(PDF)

Figure S3 Cooperative thin filament activation kinetics slow the rate of thin filament activation. The rate of thin filament activation (*k_TF,act_*) is plotted against pCa, illustrating that kinetic forms of cooperativity considerably slow thin filament activation compared to simulations with no cooperative thin filament activation kinetics. In the presence of cooperative kinetics, XB binding also increased *k_TF,act_*. In the absence of cooperative kinetics, XB binding had a minimal effect on *k_TF,act_* (inset). All simulations used standard parameter values of *k_xb_* = 3 pN nm^−1^, *k_fil_* = *k_m_* = *k_a_* = 1X, and *RUspan* = 9 actins.
(PDF)

Figure S4 Model sensitivities to cooperative parameters *Ψ* and *ξ*. Values from 3 parameter Hill fits to steady-state force-pCa relationships demonstrate the influence of *Ψ* and *ξ* on cooperativity (*n_H_*, the top set of panels), Ca^2+^ sensitivity (*pCa_50_*, the middle set of panels), and the maximal rate of force development (*k_dev_*, the bottom set of panels). All simulations used standard parameter values of *k_xb_* = 3 pN nm^−1^, *k_fil_* = *k_m_* = *k_a_* = 1X, *RU_span_* = 9 actins. All possible forms of cooperativity were implemented as *Ψ* varied from 1 to 1000, with *ξ* fixed at 100 (A–C). All possible forms of cooperativity were implemented as *Ψ* varied from 1 to 2000, with *ξ* = *Ψ* for each simulation (D–F). RU-RU and XB-XB cooperativity were implemented as *Ψ* varied from 1 to 2000, with *ξ* = *Ψ* for each simulation (G–I). Error bars represent 95% confidence intervals on the fitted parameter values for *n_H_* and *pCa_50_*, and SE for *k_dev_*.
(PDF)

Text S1 Although many of our findings were discussed in the primary manuscript, additional simulation results described in Text S1 provide a more complete picture of cooperative thin filament activation kinetics on the force-pCa and fraction available-pCa relationships (Figure S2), the rate of thin filament activation (*k_TF,act_*, Figure S3), and range of cooperative force-pCa relationships as *Ψ* and *ξ* co-varied (Figure S4).
(PDF)
