# Supplementary material for: Filament Compliance Influences Cooperative Activation of Thin Filaments and the Dynamics of Force Production in Skeletal Muscle
Source: PLoS Comput Biol. 2012 May 10;8(5):e1002506. doi: 10.1371/journal.pcbi.1002506 (PMC3349719; doi:10.1371/journal.pcbi.1002506)
Supplement: Figure S1 — Vertebrate striated muscle structure of a half-sarcomere. Computational algorithms of this spatially-explicit model represent thick filaments and thin filaments of half-sarcomere length from the M-line to the Z-line (A, color scheme consistent with Figure 1). These filaments are organized in the hexagonal lattice structure consistent with vertebrate striated muscle, more obviously demonstrated by a cross-sectional view of the A-band (B). Each filament also consists of a helical pitch describing myosin cross-bridges (XBs) extending from the thick filament or the intertwined filamentous actin helices, along which the thin filament regulatory proteins are located (C). Our computational representation of myofilament lattice organization accounts for these structural components of the muscle. (PDF) [file pcbi.1002506.s001.pdf]

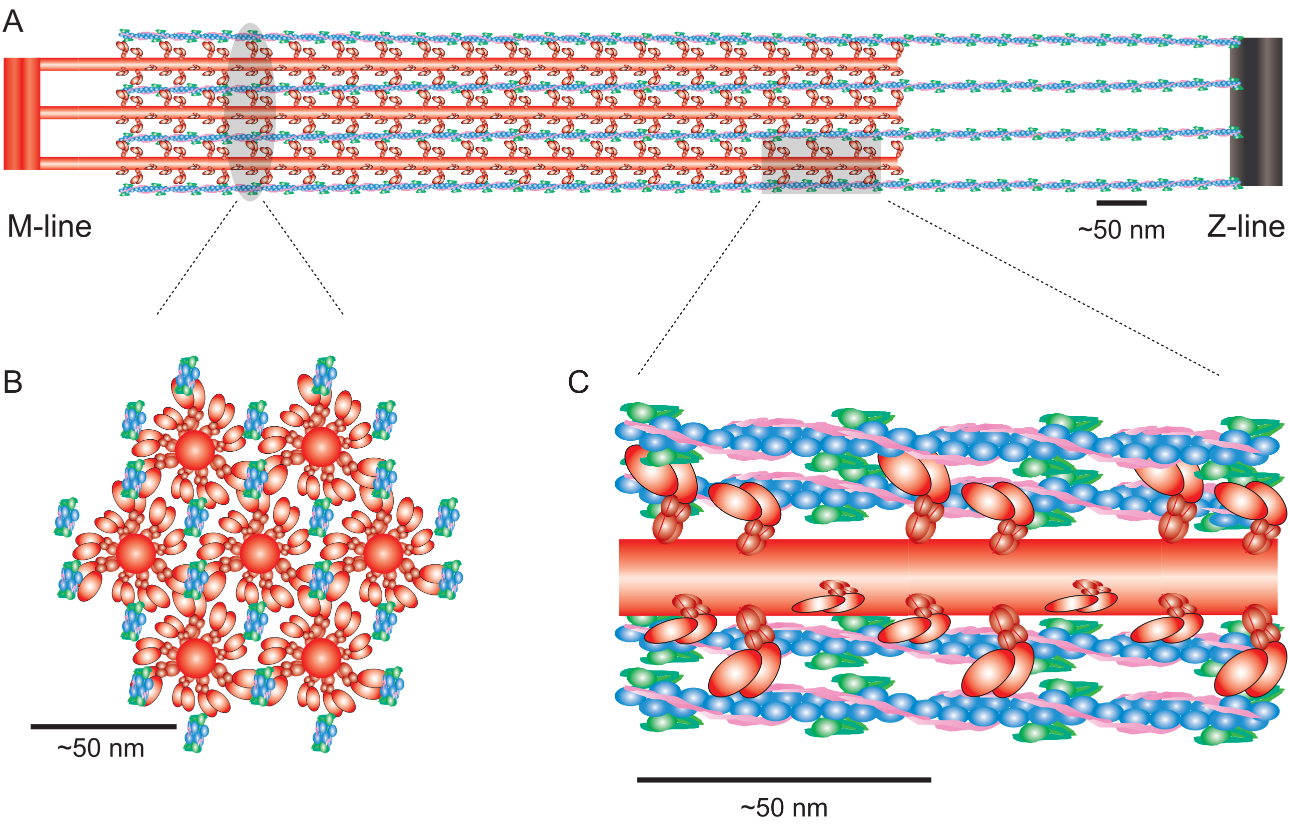

Figure S1: Vertebrate striated muscle structure of a half-sarcomere. Computational algorithms of this spatially-explicit model represent thick filaments and thin filaments of half-sarcomere length from the M-line to the Z-line (A, color scheme consistent with Figure 1). These filaments are organized in the hexagonal lattice structure consistent with vertebrate striated muscle, more obviously demonstrated by a cross-sectional view of the A-band (B). Each filament also consists of a helical pitch describing myosin cross-bridges (XBs) extending from the thick filament or the intertwined filamentous actin helices, along which the thin filament regulatory proteins are located (C). Our computational representation of myofilament lattice organization accounts for these structural components of the muscle.
