# Supplementary material for: Filament Compliance Influences Cooperative Activation of Thin Filaments and the Dynamics of Force Production in Skeletal Muscle
Source: PLoS Comput Biol. 2012 May 10;8(5):e1002506. doi: 10.1371/journal.pcbi.1002506 (PMC3349719; doi:10.1371/journal.pcbi.1002506)
Supplement: Figure S2 — Multiple cooperative pathways combine to produce the physiological force-pCa relationship. Sensitivities of force and fractional thin filament activation are illustrated for all combinations of kinetic cooperativity. (A) Lines show 3-parameter Hill fits to steady-state force-pCa responses for rt,12 being targeted independently, along with symbols depicting nH (B) and pCa50 (C) values from fits to both force-pCa and fraction available-pCa relationships. Comparable simulation results are shown for rt,23 being targeted independently (D–F), or rt,12 and rt,23 being targeted in combination (G–I). Dashed lines illustrate simulation results in the absence of cooperative kinetics. Error bars represent 95% confidence intervals on the fitted parameter values for all panels. All simulations used standard parameter values of kxb = 3 pN nm−1, kfil = km = ka = 1X, and RUspan = 9 actins, combining all possible forms of cooperativity. (PDF) [file pcbi.1002506.s002.pdf]

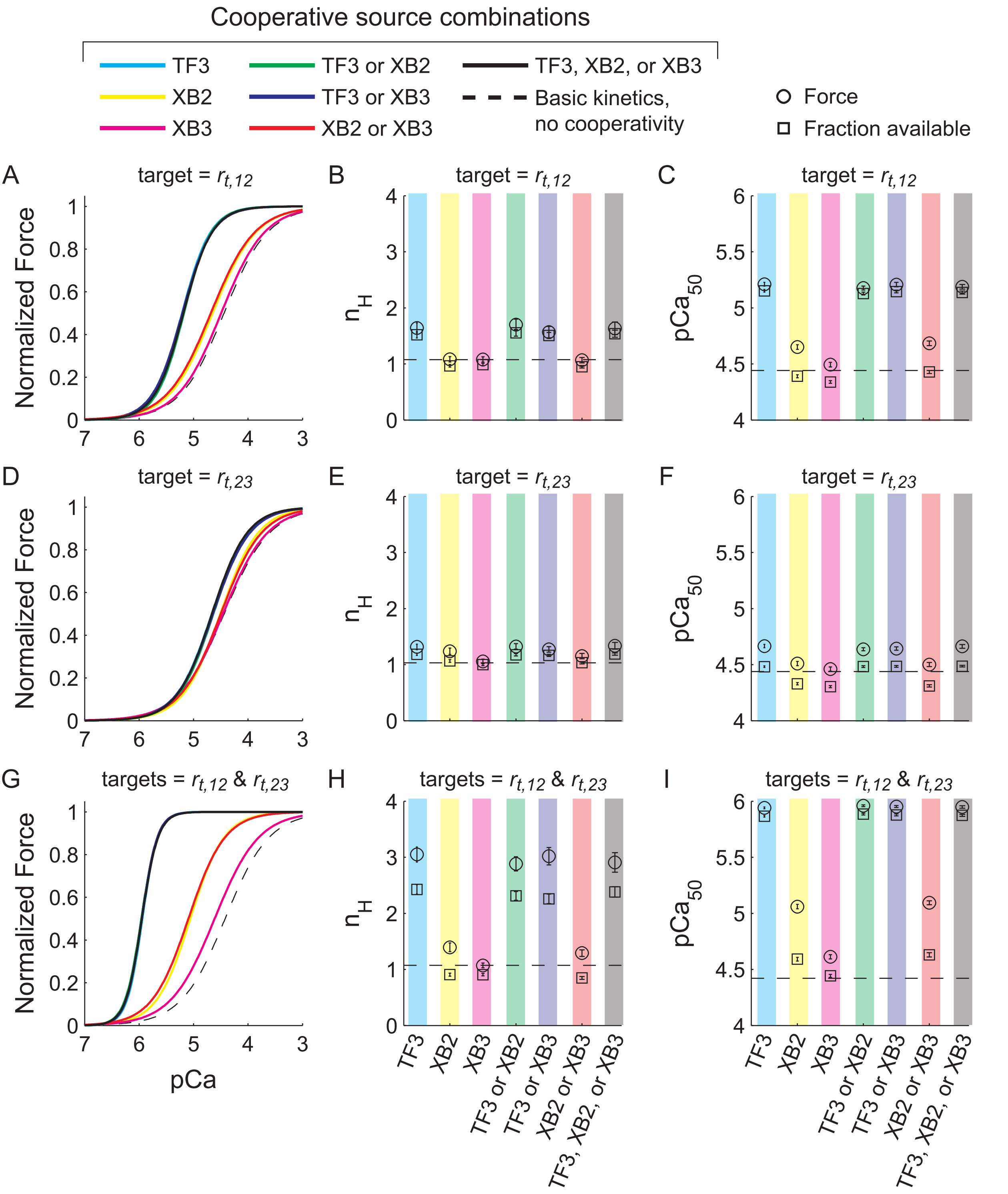

Figure S2: Multiple cooperative pathways combine to produce the physiological force-pCa relationship. Force and fractional thin filament activation are illustrated for all combinations of kinetic cooperativity. (A) Lines show 3-parameter Hill fits to steady-state force-pCa responses for $r_{t,12}$ being targeted independently, along with symbols depicting n_H (B) and pCa_{50} (C) values from fits to both force-pCa and fraction available-pCa relationships. Comparable results are shown for $r_{t,23}$ being targeted independently (D-F), or $r_{t,12}$ and $r_{t,23}$ being targeted in combination (G-I). Dashed lines illustrate simulation results without cooperative kinetics. Error bars represent 95% confidence intervals for n_H and pCa_{50}. Simulations used standard parameter values: k_{xb}=3 pN nm^{-1}, k_{fil}=k_m=k_a=1X, and RU_{span}=9 actins.
