# Supplementary material for: Filament Compliance Influences Cooperative Activation of Thin Filaments and the Dynamics of Force Production in Skeletal Muscle
Source: PLoS Comput Biol. 2012 May 10;8(5):e1002506. doi: 10.1371/journal.pcbi.1002506 (PMC3349719; doi:10.1371/journal.pcbi.1002506)
Supplement: Figure S3 — Cooperative thin filament activation kinetics slow the rate of thin filament activation. The rate of thin filament activation (kTF,act) is plotted against pCa, illustrating that kinetic forms of cooperativity considerably slow thin filament activation compared to simulations with no cooperative thin filament activation kinetics. In the presence of cooperative kinetics, XB binding also increased kTF,act. In the absence of cooperative kinetics, XB binding had a minimal effect on kTF,act (inset). All simulations used standard parameter values of kxb = 3 pN nm−1, kfil = km = ka = 1X, and RUspan = 9 actins. (PDF) [file pcbi.1002506.s003.pdf]

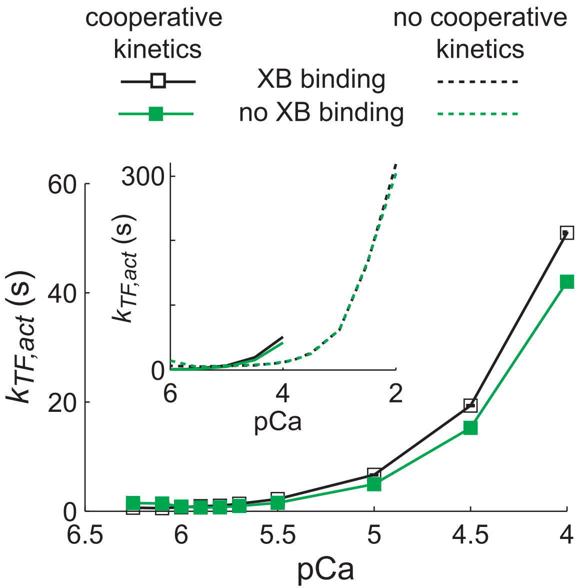

Figure S3: Cooperative thin filament activation kinetics slow the rate of thin filament activation. The rate of thin filament activation ($k_{TF,act}$) is plotted against pCa, illustrating that kinetic forms of cooperativity considerably slow thin filament activation compared to simulations without cooperative thin filament activation kinetics. In the presence of cooperative kinetics, XB binding also increased $k_{TF,act}$. In the absence of cooperative kinetics, XB binding had a minimal effect on $k_{TF,act}$ (inset). Simulations used standard parameter values: k_{xb}=3 pN nm^{-1}, k_{fil}=k_m=k_a=1X, and RU_{span}=9 actins.
