# Supplementary material for: Filament Compliance Influences Cooperative Activation of Thin Filaments and the Dynamics of Force Production in Skeletal Muscle
Source: PLoS Comput Biol. 2012 May 10;8(5):e1002506. doi: 10.1371/journal.pcbi.1002506 (PMC3349719; doi:10.1371/journal.pcbi.1002506)
Supplement: Figure S4 — Model sensitivities to cooperative parameters Ψ and ξ. Values from 3 parameter Hill fits to steady-state force-pCa relationships demonstrate the influence of Ψ and ξ on cooperativity (nH, the top set of panels), Ca2+ sensitivity (pCa50, the middle set of panels), and the maximal rate of force development (kdev, the bottom set of panels). All simulations used standard parameter values of kxb = 3 pN nm−1, kfil = km = ka = 1X, RUspan = 9 actins. All possible forms of cooperativity were implemented as Ψ varied from 1 to 1000, with ξ fixed at 100 (A–C). All possible forms of cooperativity were implemented as Ψ varied from 1 to 2000, with ξ = Ψ for each simulation (D–F). RU-RU and XB-XB cooperativity were implemented as Ψ varied from 1 to 2000, with ξ = Ψ for each simulation (G–I). Error bars represent 95% confidence intervals on the fitted parameter values for nH and pCa50, and SE for kdev. (PDF) [file pcbi.1002506.s004.pdf]

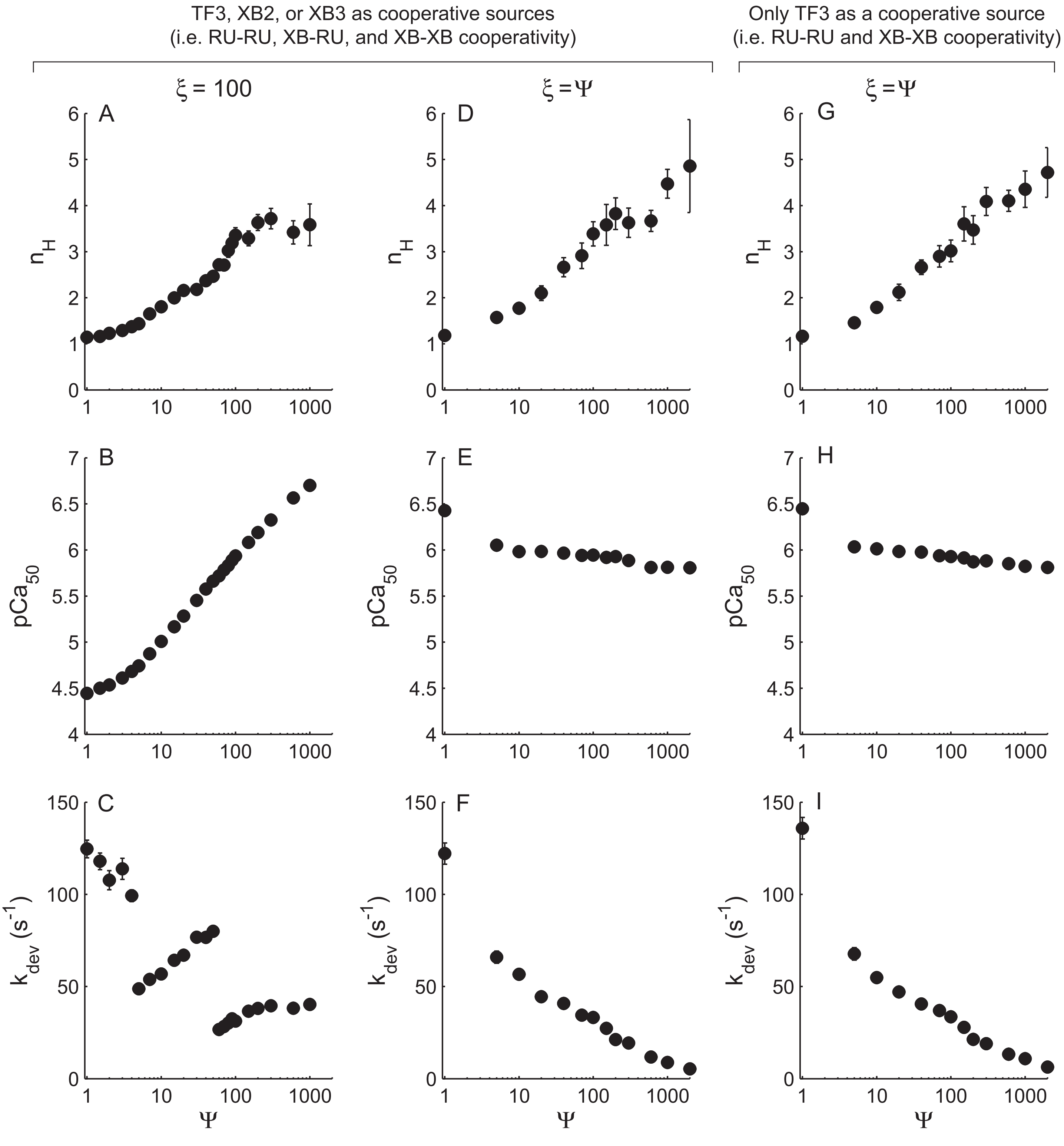

Figure S4: Model sensitivities to cooperative parameters Ψ and ξ. Values from 3 parameter Hill fits to steady-state force-pCa relationships demonstrate the influence of Ψ and ξ on cooperativity (n_H, top panels), Ca^{2+} sensitivity (pCa_{50}, middle panels), and the maximal rate of force development (k_{dev}, bottom panels). All possible forms of cooperativity were implemented as Ψ varied from 1 to 1000, with ξ fixed at 100 (A-C). All possible forms of cooperativity were implemented as Ψ varied from 1 to 2000, with ξ=Ψ for each simulation (D-F). RU-RU and XB-XB cooperativity were implemented as Ψ varied from 1 to 2000, with ξ=Ψ for each simulation (G-I). Simulations used standard parameter values: k_{xb}=3 pN nm^{-1}, k_{fil}=k_m=k_a=1X, and RU_{span}=9 actins. Error bars represent 95% confidence intervals for n_H and pCa_{50}, and SE for k_{dev}.
