# Supplementary material for: Filament Compliance Influences Cooperative Activation of Thin Filaments and the Dynamics of Force Production in Skeletal Muscle
Source: PLoS Comput Biol. 2012 May 10;8(5):e1002506. doi: 10.1371/journal.pcbi.1002506 (PMC3349719; doi:10.1371/journal.pcbi.1002506)
Supplement: Text S1 — Although many of our findings were discussed in the primary manuscript, additional simulation results described in Text S1 provide a more complete picture of cooperative thin filament activation kinetics on the force-pCa and fraction available-pCa relationships (Figure S2), the rate of thin filament activation (kTF,act, Figure S3), and range of cooperative force-pCa relationships as Ψ and ξ co-varied (Figure S4). (PDF) [file pcbi.1002506.s005.pdf]

Text S1

Effects of different sources of cooperativity on the force-pCa relationship

Figure S2 depicts the complete set of cooperative force-pCa relationships simulated for all kinetic forms of cooperativity, as well as the n_H and pCa_{50} values from 3-parameter Hill fits to these force-pCa and associated fraction available-pCa data. Consistent with the data presented in Figure 3, the relative sensitivities to cooperative sources remained TF3>XB2>XB3. In addition to Figure 3, Figure S2 illustrates the cooperative responses when $r_{t,12}$ was targeted alone (Figure S2 A-C), when $r_{t,23}$ was targeted alone (Figure S2 D-F), and when $r_{t,12}$ or $r_{t,23}$ were targeted in combination (Figure S2 G-I).

Cooperativity slows apparent rates of thin filament activation and force development

Cooperative thin filament activation kinetics significantly affect Ca^{2+} sensitivity (Figure 4) and the rate of thin filament activation ($k_{TF,act}$) whether or not XB binding was possible (Figure S3). As discussed for Figure 4, XB binding had a minimal influence on Ca^{2+} sensitivity of thin filament activation and $k_{TF,act}$ when kinetic forms of cooperativity were absent. In addition there were minimal XB-dependent differences in $k_{TF,act}$ as a function of pCa in the absence of cooperativity (Figure S3 inset), where the $k_{TF,act}$ increased non-linearly across the entire pCa range from 0 to >300 s^{-1}. In contrast, XB binding increased fractional thin filament activation in the presence of cooperativity, particularly at submaximal pCa levels (Figure 4A). XB binding also increased $k_{TF,act}$ at higher $[Ca^{2+}]$ when cooperative thin filament activation kinetics were implemented (Figure S3), ranging from 0 to 51.0±0.2 s^{-1} in the presence of XB binding versus 0 to 42.0±0.2 s^{-1} in the absence of XB binding. These cooperative $k_{TF,act}$ values are much closer to physiological values of 5 to >25 s^{-1} than $k_{TF,act}$ when kinetic forms of cooperativity were removed.

Additional data presented in Figure 5 illustrates a similar, non-linear response for the rates of force development (k_{dev}). Together, these data (Figure S4 and Figure 5 B, E, and H) show that kinetic and mechanical forms of cooperativity dramatically slow the rates of activation and force development, largely because XB-RU and XB-XB cooperativity act more slowly than RU-RU cooperativity. This prolongs the time required to reach steady-state [1] within a system of compliant filaments [
2], because XB-RU and XB-XB cooperativity depend upon XB recruitment, which continues to increase as force develops.

Experimentally, the measured rate of force redevelopment (k_{tr}) produces a non-linear, sigmoidal response as $[Ca^{2+}]$ increases, reaching a maximal value of 5 to 25 s^{-1} above pCa values of 4.8-4.5, depending upon muscle type and species [3-5]. Thus, the kinetic predictions from our model show two potential limitations compared to experimental k_{tr}: $k_{TF,act}$ and k_{dev} continually increase with increasing $[Ca^{2+}]$ and maximal values of these rates are roughly twice as fast as physiologically observed k_{tr} values. However, $k_{TF,act}$ and k_{dev} more accurately reflect measurements of force development (k_{act}) from step changes in $[Ca^{2+}]$ with caged-Ca^{2+} measurements or rapid solution switching with myofibril measurements [6, 7] than the measurements of k_{tr}, where Ca^{2+} binding to thin filaments is at (or near) steady-state. This

suggests that our kinetic rate constants for thin filament activation (Table 3) and XB binding (Figure 6) may be too large, which could make $k_{TF,act}$ and k_{dev} too fast and may explain why $k_{TF,act}$ and k_{dev} don't saturate at high [Ca^{2+}]. However, these values were scaled to reflect thin filament and XB rate constants that simulate physiological steady-state force-pCa and ATPase-pCa relationships [8], suggesting that force development may be limited by Ca^{2+}-dependent thin filament activation within skeletal muscle fibers.

Model sensitivities to ξ and Ψ

A number of preliminary simulations explored a broad set of ξ and Ψ values and many potential mechanisms of kinetic cooperativity. For instance, we tried a near-exhaustive set of simulations increasing the strength of the cooperative pathways (Ψ>1) and reducing the strength of cooperative pathways (Ψ<1), as well as potential source-target combinations of 'positive cooperativity' targeting forward activation rates $r_{t,12}$ and/or $r_{t,23}$ and 'negative cooperativity' targeting reverse activation rates $r_{t,21}$ and/or $r_{t,32}$. The simulations presented in the main body of the text represent a relatively small set of parameters that produced cooperative force-pCa relationships consistent with measurements from our laboratory.

While Ψ and ξ values of 100 may appear relatively extreme, the results shown in Figure S4 demonstrate the range of model sensitivities as Ψ and ξ varied between 1-2000. As Ψ increased from 1-1000 with ξ fixed at 100 and all forms of cooperativity implemented, n_H consistently increased from ≈1 to plateau near ≈3.6 above Ψ values of 200 (Figure S4 A). Similar to the results shown in Figure 3, however, this simulation algorithm produced large, consistent increases in pCa_{50} as Ψ increased with ξ being a fixed value (Figure S4 B). This continual shift in the pCa_{50} value with each simulation required constant adjustment of the 10 pCa values used to simulate a steady-state force-pCa relationship. Therefore, the pCa values used for the simulation results presented in Figure S4 A-C increased as Ψ increased. Specifically, the greatest [Ca^{2+}] was pCa=2 when Ψ ranged from 1 to 4, pCa=3 when Ψ ranged from 5 to 50, and pCa=4 when Ψ ranged from 60 to 1000, which led to the relatively disjoint decrease in the maximal rate of force development (k_{dev}) as Ψ increased (Figure S4 C).

As Ψ increased from 1-2000 with ξ=Ψ and all forms of cooperativity implemented, n_H consistently increased from ≈1 with a brief plateau near ≈3.6 when Ψ values ranged between 200-600 (Figure S4 D). At Ψ values of 1000 and 2000, n_H continued to increase, although uncertainty in n_H increased and simulations became increasingly unstable at the greatest Ψ values. Co-varying ξ and Ψ led to a more stable estimate of pCa_{50} across the entire range of simulations, with pCa_{50} slightly decreasing as Ψ increased (Figure S4 E). The elevated stability of pCa_{50} afforded a single set of 10 pCa values that robustly described the force-pCa relationship across all simulations. Thus, there was a more consistent decrease in k_{dev} as Ψ increased (Figure S4 F), compared to the previous set of simulations (Figure S5 C). Simulations where Ψ increased from 1-2000 with ξ=Ψ and only RU-RU kinetic cooperativity implemented (Figure S5 G-I) showed minimal differences from simulations where all forms of kinetic cooperativity were implemented (Figure S5 D-F).

This sensitivity analysis for Ψ and ξ demonstrates that cooperativity (n_H) of the force-pCa curve consistently increases with Ψ and did not saturate, although we expect that it ultimately would.

Across all of these simulations the model became relatively unstable above Ψ values of 600. Moreover, we needed to simulate force traces 40 s long to reach steady state at mid-pCa levels at, or above, Ψ values of 600. This 40 s duration is roughly ten times as long as the time-courses required to reach steady-state for simulations when Ψ was less than 600. Therefore, simulations at the highest Ψ values became increasingly intensive to perform, from the perspective of computational cost. Another consistent finding from the sensitivity analysis was the relatively large decreases in k_{dev} with increasing values of Ψ. This finding is consistent with the sub-set of simulations discussed in the main text, where k_{dev} decreases as effective cooperativity in the model increases.

The unique results provided by this spatially-explicit model represents a powerful computational paradigm that comes with significant computational costs compared to other computational models of cooperative muscle contraction. For example, each data point within a single panel of Figure S4 represents fitted parameters from a force-pCa curve composed of 10 pCa values. Each force-pCa curve took ≈ 900 CPU minutes to complete, on average, distributed across an IBM Bluemoon cluster with roughly 3,000 compute cores. For reference, the set of results shown in Figure S4 represents roughly 800 CPU hours of computation, or just over 1 CPU month. These levels of compute time can represent a significant barrier for many laboratories and may limit the practical capacity for others to utilize and improve upon this spatially-explicit computational paradigm. As discussed in the main body of the text, a number of opportunities remain available for building on and improving current models of cooperative muscle contraction, both physiologically and computationally.

Text S1 References

1. Campbell K (1997) Rate constant of muscle force redevelopment reflects cooperative activation as well as cross-bridge kinetics. Biophys J 72: 254-262.
2. Campbell K (2006) Filament compliance effects can explain tension overshoots during force development. Biophys J 91: 4102-4109.
3. Moreno-Gonzalez A, Gillis T, Rivera A, Chase P, Martyn D, et al. (2007) Thin-filament regulation of force redevelopment kinetics in rabbit skeletal muscle fibres. J Physiol 579: 313-326.
4. Brenner B (1988) Effect of Ca^{2+} on cross-bridge turnover kinetics in skinned single rabbit psoas fibers: implications for regulation of muscle contraction. Proc Natl Acad Sci USA 85: 3265-3269.
5. Metzger JM, Moss RL (1990) Calcium-sensitive cross-bridge transitions in mammalian fast and slow skeletal muscle fibers. Science 247: 1088-1090.
6. Kreutziger K, Piroddi N, Scellini B, Tesi C, Poggesi C, et al. (2008) Thin filament Ca^{2+} binding properties and regulatory unit interactions alter kinetics of tension development and relaxation in rabbit skeletal muscle. J Physiol 586: 3683-3700.
7. Tesi C, Colomo F, Piroddi N, Poggesi C (2002) Characterization of the cross-bridge force-generating step using inorganic phosphate and BDM in myofibrils from rabbit skeletal muscles. J Physiol 541: 187-199.
8. Tanner BCW, Daniel TL, Regnier M (2007) Sarcomere lattice geometry influences cooperative myosin binding in muscle. PLoS Comput Biol 3: e115.
